# Multi-Target Therapeutic Potential of Arctii Fructus Lignans in Diabetes Mellitus and Its Complications: A Mechanistic Review

**DOI:** 10.3390/ph18101569

**Published:** 2025-10-17

**Authors:** Shuaiyi Lv, Jieming Li, Yulong Hu, Juntao Cai, Guanglei Nan, Yuanfang Kong, Xu Shen, Lifeng Zhu, Shaohua Yang, Chunhong Dong

**Affiliations:** 1 Traditional Chinese Medicine (Zhong Jing) School, Henan University of Chinese Medicine, Zhengzhou 450046, China; lllshuaiyi@163.com (S.L.);; 2 Henan Key Laboratory of Chinese Medicine for Polysaccharides and Drugs Research, Zhengzhou 450046, China; 3 Xinxiang Tuoxin Pharmaceutical Co., Ltd., Xinxiang 453000, China

**Keywords:** *Arctium lappa* L., lignans, diabetes mellitus, diabetes mellitus complications

## Abstract

This narrative review systematically evaluates the therapeutic potential and mechanisms of action of Arctii Fructus lignans (from *Arctium lappa* L.) in managing diabetes mellitus (DM) and its complications. To ensure a comprehensive evidence synthesis, a systematic literature search was conducted using PubMed, Web of Science, CNKI, and Wanfang Data for publications between January 2000 and June 2024. Study selection was based on predefined inclusion and exclusion criteria focusing on lignan-specific antidiabetic effects and mechanistic insights. The accumulated evidence demonstrates that Arctii Fructus lignans exhibit multi-targeted pharmacological effects through several key mechanisms: (1) improving glucose homeostasis via α-glucosidase inhibition and *AMPK/PI3K* pathway activation; (2) protecting pancreatic β-cell function through anti-inflammatory and anti-apoptotic actions; and (3) mitigating diabetic complications by reducing oxidative stress, modulating *TGF-β/VEGF* signaling, and restoring autophagy balance. Notably, these lignans show particular efficacy in early-stage diabetes models, with reduced effectiveness in advanced stages featuring significant β-cell dysfunction, suggesting a critical therapeutic window for intervention. Future research should prioritize well-designed clinical trials using standardized extracts, investigations into structure–activity relationships, and exploration of synergistic effects within traditional formulations to advance the translational potential of these promising natural compounds.

## 1. Introduction

The global burden of diabetes mellitus (DM) is rising at an alarming pace. According to the International Diabetes Federation, the number of individuals affected is projected to reach 783 million by 2045, marking a 46% increase over the next two decades. Persistent hyperglycemia in diabetic patients contributes to progressive multiorgan dysfunction through continuous metabolic disturbances, ultimately resulting in microvascular and macrovascular complications. These include cardiovascular diseases, peripheral neuropathy, retinopathy, and nephropathy [1]. The complexity and growing prevalence of these complications underscore DM as a major public health concern with significant global healthcare implications.

**Figure 1:**
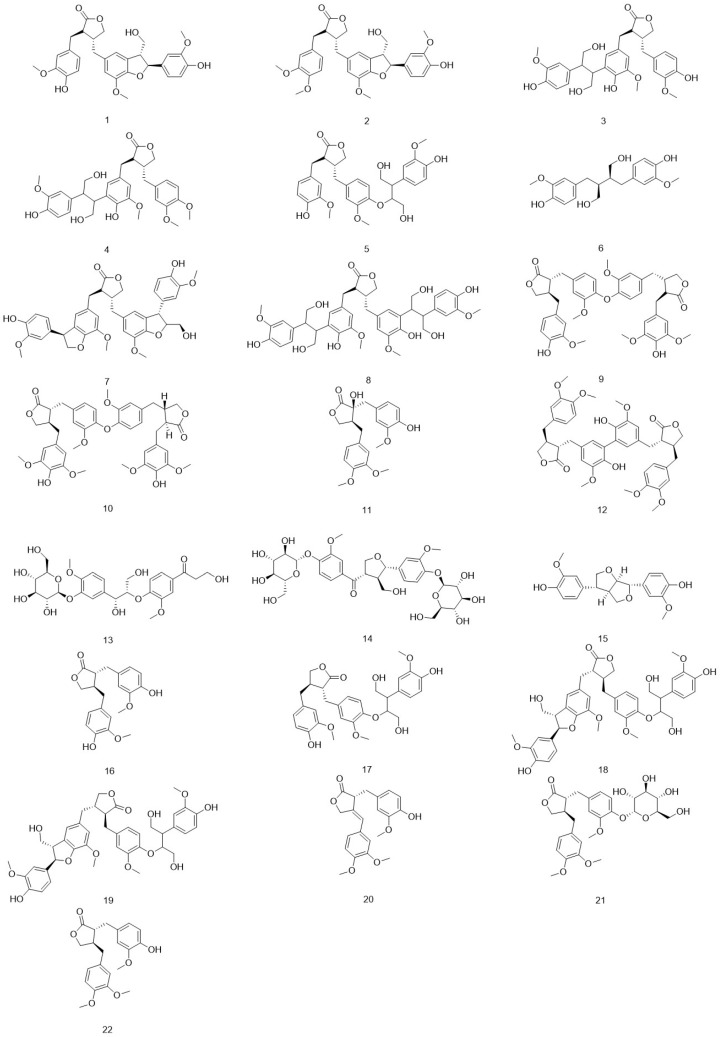
Chemical structures of the main lignans from *Arctium lappa* L.

Arctii Fructus (*Arctium lappa* L., Asteraceae), a biennial medicinal plant, is well-documented in the pharmacopeia of traditional Chinese medicine (TCM); its medicinal part specifically refers to the dried ripe fruits of this plant, which can be used as a medicinal herb after being processed by stir-frying [2], the Chinese Pharmacopoeia stipulates that the theoretical plate number in Codonopsis seeds should be no less than 1500 when calculated based on the Codonopsis glycoside peak. It serves as a key therapeutic constituent in several antidiabetic remedies, including the classical “Xiao Ke Tea” (recorded in Qian Jin Yao Fang, Arctii Fructus 15 g; Rehmanniae Radix 12 g; Glycyrrhizae Radix 6 g, decocted) and modern formulations such as Sheng Jin Yu Ye capsules (0.3 g Arctii Fructus lignan extract per capsule) and Yu Xiao powder (Cortex Lycii (Digu Pi): 30 g; Semen Litchi (Lizhi He): 10 g; Ramulus Euonymi (Guijian Yu): 45 g; Radix Platycodi (Jiegeng): 15 g; Radix Clematidis (Weilingxian): 10 g; Rhizoma Curcumae Longae (Jianghuang): 10 g; and Fructus Arctii (Niubangzi): 10 g), which are used in the management of type 2 diabetes [3,4]. Its hypoglycemic effects have been the subject of extensive research for several decades. Cui et al. (1997) first reported the dual therapeutic potential of Arctii Fructus in reducing hyperglycemia and improving renal protein clearance in clinical observations [5]. While this study provided initial clinical hints, it lacked rigorous phytochemical characterization of the test material, making it difficult to attribute the observed effects specifically to lignans. Therefore, its findings should be interpreted with caution. Nonetheless, this early signal has motivated subsequent modern preclinical investigations, which have robustly delineated the glucoregulatory mechanisms of purified lignans (as discussed in Section 3.1 and Section 3.2), thereby providing a mechanistic plausibility for the earlier clinical observation. Later preclinical investigations confirmed the therapeutic potential of the ethanol extract of Arctii Fructus in streptozotocin (STZ)-induced diabetic murine models, revealing both antihyperglycemic and immunomodulatory activities [2]. These preclinical outcomes are further supported by clinical data, wherein Wu et al. (1997) documented significant therapeutic efficacy in type 2 diabetes patients administered Arctii Fructus as a monotherapy [4]. Integrated phytochemical and pharmacological investigations have provided insights into the antidiabetic mechanisms of Arctii Fructus. Xu et al. (2005) reported that ethanol extracts demonstrated significantly higher α-glucosidase inhibitory and hypoglycemic activities compared to aqueous extracts, a difference attributed to the presence of lipophilic constituents [6]. Chemical profiling has identified several bioactive constituents, including lignans, cinnamic acid derivatives, and sesquiterpene lactones. Among these, lignans represent the principal pharmacologically active class [7]. The pharmacological diversity of Arctii Fructus lignans is underpinned by their chemical structural diversity. The most prevalent lignans, such as arctiin (a glycoside) and its aglycone arctigenin, share a core dibenzylbutyrolactone skeleton. Critical variations in functional groups, particularly the presence or absence of glycosidic moieties, hydroxylations, and methoxylations, profoundly influence their bioavailability, metabolic fate, and biological targets [8]. For instance, the glycosidic form (arctiin) may have different absorption properties compared to the aglycone (arctigenin), which can be liberated by gut microbiota, leading to varied onset and duration of action. This structure–activity relationship (SAR) is crucial yet not fully elucidated [8]. Particularly, arctiin and arctigenin, two predominant lignan derivatives, have received particular attention for their multi-target modulatory effects on diabetic pathophysiology and its related complications [9,10]. The identified lignans are presented in Figure 1 and Table 1. Therefore, the primary objectives of this narrative review are fourfold: (1) to systematically consolidate and evaluate the preclinical evidence supporting the multi-targeted therapeutic effects of Arctii Fructus lignans against DM and its complications; (2) to critically discuss the molecular mechanisms underlying their efficacy, with a focus on structure–activity relationships where evidence permits; (3) to identify and analyze the existing research gaps, such as inconsistencies in experimental models and the limited clinical translation; and (4) to provide a critical perspective on the future research directions necessary to advance these natural compounds towards clinical application.

## 2. Therapeutic Roles of Arctii Fructus Lignans in Diabetes Mellitus

DM is a common endocrine disorder categorized into two primary types: type I insulin-dependent DM (IDDM) and type II noninsulin-dependent DM (NIDDM) [24]. Type I DM is an autoimmune disorder characterized by immune-mediated destruction of pancreatic β-cells, leading to insulin deficiency and persistent hyperglycemia [25]. Type II DM, representing over 90% of cases, is driven by a combination of peripheral insulin resistance and β-cell dysfunction, resulting in both fasting and postprandial glucose dysregulation [26]. The therapeutic potential of Arctii Fructus in diabetes management has been clinically recognized within TCM, while modern pharmacognostic studies have identified lignans as the primary bioactive constituents and elucidated their underlying mechanisms of action.

### 2.1. Efficacy of Arctii Fructus lignans in Different Diabetes Models

As shown in Table 2, preclinical studies have demonstrated that Arctii Fructus lignans exert significant glycemic regulatory effects across various diabetic models, including alloxan-induced type II diabetic mice, high-fat diet/STZ (HFD/STZ)-treated rats, spontaneous diabetic GK rats, obese KKAy mice, db/db mice, and STZ-induced type I diabetic models. These interventions consistently normalized fasting glucose, improved postprandial glucose tolerance, and alleviated dyslipidemia, as reflected by decreased TG and TC levels and increased HDL-C concentrations [7,27,28,29,30]. Model-specific differences were observed: GK rats (nonobese type II diabetic models) showed no lignan-induced lipid modulation, indicating the need for further investigation to clarify the underlying reasons for this model-specific variation.

Investigations using KKAy *mice* (a spontaneous obese type II diabetic model) confirmed the lipid-regulating properties of lignans, highlighting model-specific responsiveness associated with distinct metabolic phenotypes [29,30]. These findings demonstrate that Arctii Fructus lignans effectively lower blood glucose, increase insulin secretion, and improve dyslipidemia in type I and type II diabetic models, although their effect on LDL-C appears to be context-dependent. A reduction in LDL-C was observed only in C57BL/6J mice subjected to HFD/STZ induction, whereas no significant effect was observed in GK or db/db models, suggesting that diet-induced dyslipidemia may be a prerequisite for this activity. The context-dependent modulation of LDL-C by Arctii Fructus lignans carries significant physiological and clinical implications. Elevated LDL-C is a well-established risk factor for cardiovascular disease (CVD), a major cause of morbidity and mortality in diabetic patients [31]. The observed reduction in LDL-C specifically in the HFD/STZ model, which mimics diet-induced dyslipidemia common in human type 2 diabetes, suggests that these lignans may exert their lipid-lowering benefits most effectively in individuals with pronounced dietary contributions to their metabolic syndrome. The lack of effect in genetic models like GK or db/db mice, which have distinct, genetically driven dyslipidemic profiles, underscores the importance of the underlying pathophysiology in determining therapeutic responsiveness. This highlights the potential for personalized approaches in utilizing Arctii Fructus lignans, where their LDL-C-lowering effect might be prioritized for diabetic patients with significant diet-induced dyslipidemia, thereby offering a targeted strategy to mitigate CVD risk. The data also indicate potential dose dependency (e.g., 350 mg/kg eliciting TG reduction in alloxan–Wistar rats versus 300 mg/kg showing no effect [7,29]), compound-specific effects (total lignans versus individual arctiin/arctigenin), and model-specific pathophysiological influences. This nonlinear dose–response relationship implies possible receptor saturation or threshold-dependent activation kinetics, warranting comprehensive pharmacokinetic–pharmacodynamic modeling.

**Table 2:** Summary of key preclinical studies on antidiabetic efficacy of Arctii Fructus Lignans. The symbol “↓” indicates a decrease in the level of the corresponding indicator, while “↑” indicates an increase in the level of the corresponding indicator.

Diabetic Model	Lignan Type	Key Antidiabetic Effects	References
STZ-induced Type 1 DM (*mouse/rat*)	Arctigenin	↓ Fasting glucose, ↑ Insulin secretion, ↓ Pancreatic oxidative stress	[7,27]
HFD/STZ-induced Type II DM (*rat*)	Total lignan	↓ Fasting glucose, ↓ TG/TC, ↑ HDL-C, ↑ *GLUT4* expression	[28,30]
Spontaneous Type II DM (db/db *mouse*)	Arctiin	↓ Postprandial glucose, ↓ Insulin resistance, ↑ Adiponectin	[29,32]
Spontaneous Type II DM (GK *rat*)	Arctigenin	↓ Fasting glucose, no significant effect on lipid profile	[30,33]
Diabetic nephropathy (STZ-induced, *rat*)	Total lignan	↓ Urinary microalbumin, ↓ Renal *TLR4/NF-κB* activation	[34]

### 2.2. Mechanisms of Arctii Fructus Lignans Against Diabetes

**Figure 2:**
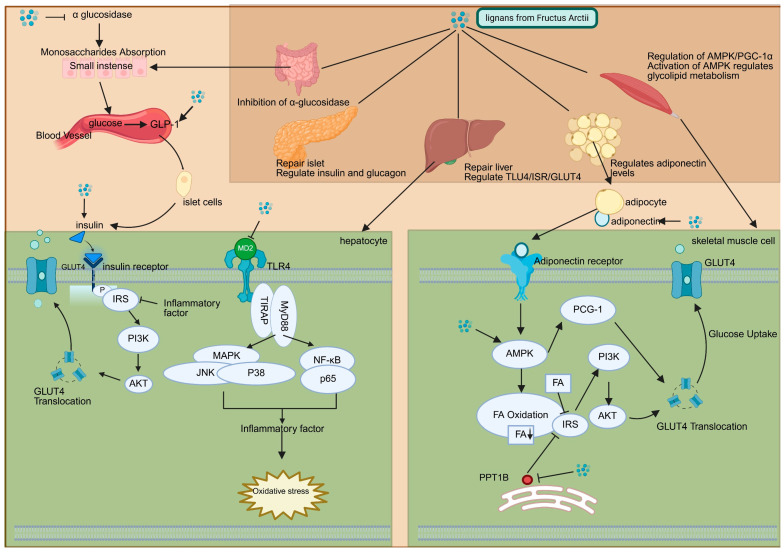
Molecular pathways involved in the antidiabetic effect of *Arctium lappa* lignans. (Created in Biorender.com: https://app.biorender.com/).

The hypoglycemic mechanisms of Arctii Fructus lignans operate through multiple pathways, as illustrated in Figure 2, including inhibition of α-glucosidase, improvement of insulin resistance, protection of pancreatic β-cells, and restoration of metabolic homeostasis. α-Glucosidase, a membrane-bound intestinal enzyme responsible for the hydrolysis of carbohydrates via cleavage of the α-glucopyranoside bond, serves as a key therapeutic target [35]. Competitive inhibition of this enzyme delays glucose absorption, reduces postprandial hyperglycemia, and alleviates pancreatic oxidative stress, thus contributing to both the prevention and management of diabetes [36]. An et al. (2013) first identified α-glucosidase inhibition as a key mechanism underlying the hypoglycemic effects of arctiin [28], a finding later supported by Xu et al., who demonstrated that the total lignan extract achieves a substantial inhibition rate of approximately 70% at a concentration of 200 μg/mL, a level of activity equivalent to that of the clinical drug acarbose at the same concentration. Furthermore, at a higher concentration of 500 μg/mL, the inhibition rate approaches 90%, indicating a potent and dose-dependent effect [29]. In vitro studies further revealed that arctigenin possesses stronger α-glucosidase inhibitory activity than its precursor, arctiin [37]. Pharmacokinetic analyses, however, indicate that intestinal microbiota-mediated biotransformation of arctiin into arctigenin via catechol-O-methyltransferase contributes significantly to in vivo efficacy [8,38]. This metabolic conversion highlights the therapeutic benefit of oral lignan administration, despite variations in compound-specific activity.

Insulin resistance, a defining feature of type II DM pathogenesis, is mitigated through multiple lignan-mediated mechanisms. Arctigenin downregulates Toll-like receptor (*TLR*) expression and suppresses *NF-κB/MAPK* signaling, reducing proinflammatory cytokine-induced disruption of *IRS-2/GLUT4* pathways and restoring insulin sensitivity [32]. Complementary findings by Gao et al. demonstrated that total lignans elevate *PI3K/GLUT4* activation and inhibit protein tyrosine phosphatase-1B (*PTP1B*), leading to upregulation of adiponectin and increased insulin signaling [30]. Furthermore, activation of AMP-activated protein kinase (AMPK) via the *AMPK/PGC-1α* pathway has been identified as a pivotal mechanism for insulin sensitization, with arctiin showing metformin-like effects on hepatic glucose metabolism [33].

Pancreatic β-cell dysfunction, a central pathogenic factor in both DM subtypes, is ameliorated by lignan-mediated restoration of islet architecture. Histomorphometric analyses demonstrated increased β-cell proliferation, normalization of the α/β-cell ratio, and improved insulin/glucagon expression following lignan treatment [29,30]. Moreover, Zhou et al. reported that arctigenin exerts hepatopancreatic protective effects, characterized by reduced inflammatory infiltration, decreased lipid droplet accumulation, and suppression of *TLR4/MyD88/NF-κB* signaling, a pathway implicated in TNF-α- and IL-6-mediated tissue injury [32,34]. Despite these advances, the precise molecular mechanisms underlying pancreatic protection remain to be fully elucidated, warranting further investigation.

Arctii Fructus lignans have been shown to ameliorate glucose–lipid metabolic disturbances [30]. By inhibiting PTP1B and activating the PI3K/Akt signaling pathway, lignans elevate muscular glucose uptake, while adiponectin-mediated activation of AMPK facilitates hepatic fatty acid oxidation and suppresses gluconeogenesis. Huang et al. identified a novel mechanism of AMPK activation whereby arctigenin inhibits mitochondrial complex I, modulating the cellular AMP/ATP ratio independently of LKB1 (liver kinase B1), CaMKK (Ca/calmodulin-dependent protein kinases), or reactive nitrogen species pathways [39]. This metabolic reprogramming effectively ameliorates hyperglycemia and dyslipidemia without inducing hypoglycemia under normoglycemic conditions, offering an advantage over conventional agents such as glibenclamide. The glucose-dependent hypoglycemic profile of lignans resembles that of sulfonylureas [7], while their AMPK-mediated metabolic effects parallel those of metformin and thiazolidinediones [30], positioning them as multifunctional antidiabetic agents.

The complex pathophysiology of diabetes highlights the necessity for continued mechanistic investigation. Although current studies have identified several molecular targets, a comprehensive understanding of lignan–receptor interactions and associated signaling crosstalk remains incomplete. Systematic exploration of these pharmacodynamic processes holds promise for advancing our understanding of diabetes pathobiology and facilitating the development of lignan-based therapeutic strategies.

## 3. Therapeutic Roles of Arctii Fructus Lignans in Diabetic Complications

Diabetic complications include a spectrum of pathological conditions arising from chronic DM, including cardiovascular diseases, neuropathy, retinopathy, nephropathy, and other systemic disorders. These complications primarily result from persistent hyperglycemia, leading to progressive damage across multiple tissues and organs. As the global prevalence of diabetes rises, the burden of its associated complications is also increasing. This underscores the urgent need for effective therapeutic strategies. Emerging evidence indicates that lignans from Arctii Fructus possess significant potential in modulating specific diabetic complications, offering promising prospects for pharmacological research and therapeutic development.

### 3.1. Arctii Fructus lignans in Diabetic Peripheral Neuropathy

Diabetic peripheral neuropathy (DPN) is a common chronic complication of DM, affecting approximately 20% of patients with type 1 diabetes and 50% of those with type 2 diabetes [40]. It manifests through a range of symptoms, with neuropathic pain being a predominant and clinically debilitating feature. Patients often experience spontaneous pain (e.g., burning, shooting sensations), evoked pain (such as mechanical allodynia—pain from non-painful stimuli), hyperalgesia (increased pain response), tingling (paresthesia), and numbness, significantly impairing quality of life [41]. Its etiopathogenesis remains incompletely elucidated; however, current evidence implicates glucose–lipid metabolic dysregulation as the primary driver, with additional contributions from chronic inflammation, oxidative stress, and impaired autophagy flux [42]. At present, no disease-modifying pharmacotherapy is available for DPN. Conventional treatment focuses on symptomatic relief through the use of antioxidants, microcirculatory enhancers, metabolic modulators, neurotrophic agents, and analgesics, yet therapeutic efficacy remains limited.

Recent studies underscore the potential of TCM in DPN management, particularly through integrative strategies that combine TCM syndrome differentiation with contemporary pharmacological approaches, achieving improved clinical outcomes compared with monotherapies [43,44]. This integrative paradigm underscores the need for translational research to bridge TCM theory with modern technological innovations, enabling the development of mechanistically targeted therapeutics for DPN.

Chronic hyperglycemia is mechanistically linked to impaired autophagy and activation of apoptotic pathways. Pharmacological induction of autophagy exerts cytoprotective effects by reducing oxidative stress, increasing ATP synthesis, and activating anti-apoptotic mechanisms [45]. Experimental evidence indicates that arctigenin (50 mg/kg) significantly improves sciatic nerve pathology in DPN model *mice*, as demonstrated by decreased malondialdehyde (MDA) levels, restoration of glutathione (GSH) and catalase (CAT) activities, increased *Beclin-1* expression, elevated LC3-II/LC3-I ratio, and reduced p62/*SQSTM1* accumulation. These molecular alterations are accompanied by a reduced *Bax/Bcl-2* ratio and suppressed cleaved *caspase-3* expression, correlating with functional recovery manifested by attenuated mechanical allodynia, increased pain thresholds, and preserved spinal cord gray matter architecture. Mechanistically, arctigenin promotes autophagy restoration via coordinated modulation of the *PI3K/AKT/mTOR* and *AMPK/TSC2* signaling pathways, mediated through TNF-α suppression and mTOR inhibition [46].

**Figure 3:**
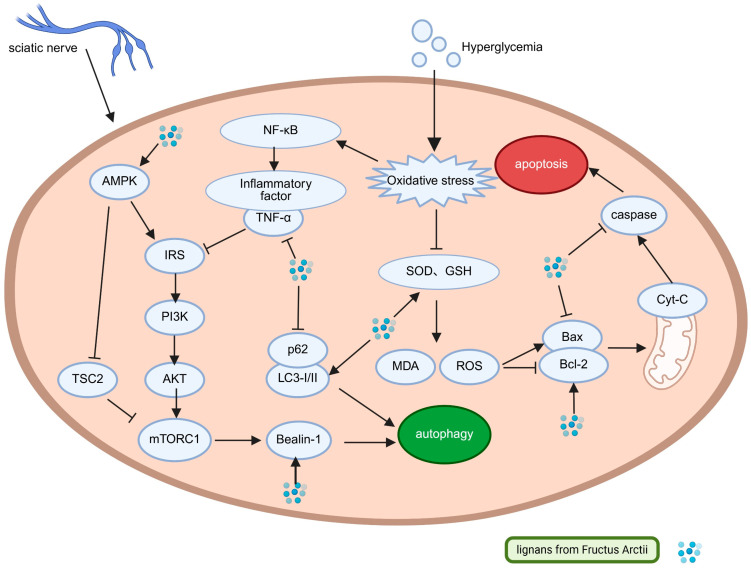
Molecular pathways involved in the antidiabetic peripheral neuropathy effect of *Arctium lappa* lignans. (Created in Biorender.com: https://app.biorender.com/).

Research gaps persist regarding the effects of Arctii Fructus lignans on DPN, especially concerning their role in modulating mitochondrial dynamics, a key pathogenic process regulated by AMPK [47]. Arctii Fructus lignans activate AMPK through two primary mechanisms: inhibition of mitochondrial complex I, leading to altered AMP/ATP ratios (metformin-like effects), and adiponectin-mediated AMPK phosphorylation. AMPK exerts pleiotropic regulatory functions in glucose–lipid homeostasis, inflammation, oxidative defense, and autophagy–apoptosis balance, all of which are central to DPN pathogenesis. These lignans display multi-target therapeutic potential, including activation of the *AMPK/Nrf2* and *AMPK/PGC-1α/PPARα* pathways (boosting antioxidant defenses) [48,49], modulation of the *AMPK/SIRT1* pathway (mediating anti-inflammatory and anti-apoptotic responses), promotion of *AMPK/ULK1*-dependent autophagy flux, and improvement of insulin sensitivity through *AMPK/PGC-1α* signaling, as illustrated in Figure 3 [39,46,50,51,52].

Systematic studies assessing the therapeutic efficacy of Arctii Fructus lignans in DPN models remain limited, despite available mechanistic insights. Although current molecular findings offer a theoretical basis, significant translational gaps remain unresolved. Future investigations should emphasize preclinical validation of their lipid-regulating properties in DPN, establish dose–response relationships for lignan fractions, compare the pharmacokinetics of arctiin and arctigenin, and clarify their pancreatic protective effects. Addressing these aspects will provide a solid framework for advancing lignan-based therapeutics with multifaceted mechanisms of action for DPN management.

### 3.2. Arctii Fructus lignans in Diabetic Nephropathy

Diabetic nephropathy (DN) is characterized by persistent proteinuria and a progressive decline in glomerular filtration rate (GFR) [53]. Hyperglycemia-induced hemodynamic disturbances, combined with metabolic alterations, synergistically impair glomerular filtration barrier (GFB) integrity, with its structural and functional deterioration being central to proteinuria development and disease progression [54]. The therapeutic effects of Arctii Fructus in DN were first reported in 1991, highlighting its capacity to reduce hyperglycemia and proteinuria [5], prompting further pharmacological investigations. Wang et al. (2003) reported varying renoprotective effects among crude powder, aqueous extract, and ethanolic extract of Arctii Fructus, with the ethanol fraction showing superior efficacy by reducing urinary microalbumin and downregulating profibrotic mediators (PAI-1, c-fos, c-jun) [55]. Phytochemical analyses later identified lignans, primarily arctiin and arctigenin, as the key bioactive constituents. Clinical validation was provided in 2011, where arctiin granules significantly decreased 24-h urinary protein excretion compared with placebo [56], highlighting their translational potential.

Mechanistic investigations reveal that Arctii Fructus lignans confer multifaceted renoprotective effects through several interconnected pathways. These include attenuation of oxidative stress (evidenced by increased renal SOD and GSH-Px activities with concomitant reductions in MDA and LPO), suppression of inflammatory signaling via *PP2A*-mediated inhibition of the *TNF-α/NF-κB* pathway, and alleviation of endoplasmic reticulum stress through downregulation of *GRP78/CHOP/caspase-12* [57,58,59,60,61]. Modulation of fibrotic progression is also achieved by disrupting the *TGF-β1/CTGF* pathway. The *TGF-β/Smad* signaling pathway plays a central role in DN-related fibrosis, wherein ligand binding triggers *Smad2/3* phosphorylation and their nuclear translocation with *Smad4*, therefore promoting excessive extracellular matrix (ECM) deposition and glomerulosclerosis [62]. CTGF is recognized as a downstream effector of TGF-β1, promoting fibrogenesis by increasing the production of α-SMA, collagen I/IV, and fibronectin [63,64]. The activity of this pathway is closely correlated with clinical biomarkers of DN progression. Elevated levels of TGF-β1 in urine or serum have been associated with declining glomerular filtration rate (GFR) and increased proteinuria, serving as indicators of active fibrotic processes [65]. Similarly, CTGF levels are often elevated in diabetic patients with nephropathy and correlate with the severity of renal fibrosis and functional impairment [66]. The inhibition of these pathways by Arctii Fructus lignans, as evidenced by reduced expression of downstream effectors like α-SMA and collagen, suggests a mechanism that may translate into amelioration of these key clinical biomarkers, potentially slowing the progression of DN. Arctigenin has been reported to mitigate these effects by concurrently inhibiting *PDGF-BB* and Vascular endothelial growth factor (*VEGF*), key mediators of critical regulators of mesangial proliferation and glomerular hyperpermeability in DN [57,67,68]. VEGF plays a significant role in DN by influencing kidney podocytes, leading to increased vascular permeability. Elevated VEGF levels are a hallmark of DN and compromise the glomerular filtration barrier, contributing to proteinuria [69]. Another key mechanism pertinent to DN progression involves podocyte preservation, where arctigenin increases actin cytoskeletal stability via *PP2A*-mediated *DBN1* dephosphorylation, reducing foot process effacement and protein leakage [69]. Moreover, the restoration of *nephrin* and *podocin* expression in DN model supports slit diaphragm integrity within the glomerular barrier [70].

**Figure 4:**
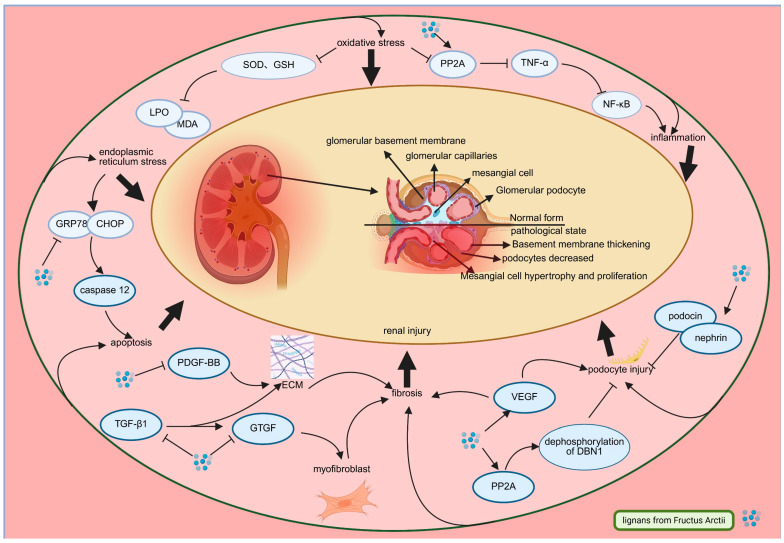
Molecular pathways involved in the antidiabetic nephropathy effect of *Arctium lappa* lignans. (Created in Biorender.com: https://app.biorender.com/).

The pathophysiological complexity of DN, involving complex signaling network crosstalk and multiorgan interactions, warrants deeper mechanistic investigation. As illustrated in Figure 4, current research on Arctii Fructus lignans has identified only isolated molecular targets, highlighting the need for systems-level analyses of pathway interconnectivity, tissue-specific pharmacokinetic characterization, and comprehensive long-term efficacy and safety assessment.

### 3.3. Arctii Fructus lignans in Diabetic Retinopathy

Diabetic retinopathy (DR) is a progressive retinal microvascular disorder and represents one of the most severe complications of DM [71]. It is the second leading cause of visual impairment worldwide, following age-related macular degeneration [72], and its prevalence is estimated to reach 191 million individuals by 2030 [73].

This complex pathology results from multifaceted pathogenic mechanisms involving microvascular dysfunction, neuronal degeneration, and chronic inflammatory signaling [74]. Clinically, DR is classified into non-proliferative (NPDR) and proliferative (PDR) stages, based on the extent of vascular impairment and ischemia-related changes [75]. Given the central roles of inflammation, vascular damage, and neurodegeneration in DR pathogenesis, therapeutic strategies targeting these pathways are of significant interest. Evidence indicates that lignans from Arctii Fructus, particularly arctiin, possess pharmacological properties relevant to these mechanisms, suggesting therapeutic potential for ophthalmic pathologies. Arctiin has been shown to cross the blood–brain barrier, implying possible ocular bioavailability. Given the well-known physiological and structural similarities between the BBB and the inner blood–retinal barrier (iBRB)—both are composed of tightly joined endothelial cells supported by pericytes and glial cells—the ability to penetrate the BBB provides a strong rationale for predicting comparable permeability across the iBRB. This inference significantly bolsters the plausibility that arctigenin (and potentially its precursor arctiin, which may be metabolized to arctigenin systemically or locally) can reach therapeutic concentrations within the retinal tissue, thereby directly mediating the observed improvements in retinal architecture and function in diabetic models [76]. Experimental studies in diabetic rodent models demonstrated that arctiin administration improved retinal architecture, characterized by increased inner nuclear layer cellularity and increased retinal thickness [77], supporting its potential role in managing diabetic ocular complications.

**Figure 5:**
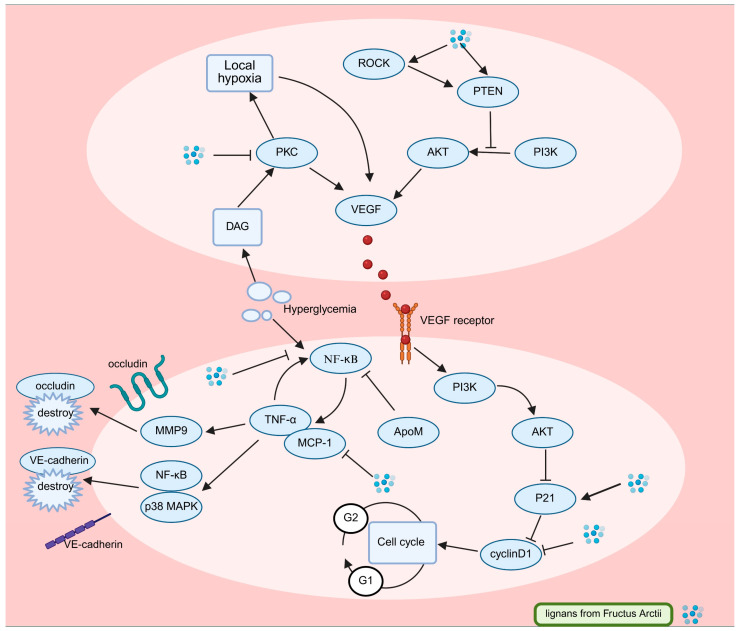
Molecular pathways involved in the antidiabetic retinopathy effect of *Arctium lappa* lignans. (Created in Biorender.com: https://app.biorender.com/).

Mechanistic studies reveal that arctiin exerts anti-DR effects through multimodal regulation of inflammatory mediators, VEGF signaling, and retinal junctional protein dynamics, as illustrated in Figure 5. Arctiin significantly reduces high glucose-induced overexpression of TNF-α, MCP-1, and *VEGF* in human retinal microvascular endothelial cells (HRCECs) while concurrently modulating cell cycle regulators by suppressing *cyclin D1* and upregulating *p21* [78]. This combined action mitigates pathological endothelial proliferation and reestablishes inflammatory balance, with apolipoprotein M (ApoM) contributing context-dependent anti-inflammatory effects under hyperglycemic conditions [79,80,81,82].

Under hyperglycemic and inflammatory conditions characteristic of DR, VEGF overexpression is a key pathogenic event. Its role evolves with disease stage: In non-proliferative DR (NPDR), VEGF primarily contributes to increased vascular permeability, leading to macular edema and vision loss. As ischemia progresses, driving the transition to proliferative DR (PDR), VEGF becomes a potent stimulator of pathological angiogenesis, characterized by the growth of fragile new blood vessels that are prone to hemorrhage and retinal detachment, posing a severe threat to sight. Collectively, these VEGF-driven processes accelerate retinal damage and DR progression [83,84,85,86]. Targeting this central mediator is therefore a crucial therapeutic strategy. Arctiin mitigates these effects by activating the *ROCK1/PTEN* pathway, which inhibits *PI3K/Akt* signaling and suppresses VEGF expression [87,88,89]. ROCK1 kinase promotes PTEN phosphorylation, strengthening its inhibitory effect on PI3K/Akt-driven angiogenic signaling [90,91]. This mechanism has been confirmed using the ROCK1 inhibitor Y-27632, which dose-dependently reduces VEGF expression [92,93,94]. In vivo evidence further supports arctiin’s anti-VEGF activity, as demonstrated by Lu et al., who reported a significant decrease in retinal VEGF levels in diabetic models [95], suggesting potential benefits across both NPDR (reducing edema) and PDR (inhibiting neovascularization) stages.

Complementary studies by Xu et al. highlighted modulation of protein kinase C (PKC) isoforms as an additional therapeutic mechanism, showing that arctiin inhibits hyperglycemia-induced diacylglycerol (DAG) accumulation and the resulting PKC activation [95,96]. Specific PKC isoforms (βI/II) play a central role in DR pathogenesis by promoting basement membrane thickening, leukocyte adhesion, and vascular hyperpermeability [97]. PKC-mediated retinal hypoperfusion generates hypoxic microenvironments that further promote VEGF expression, creating a self-amplifying pathogenic loop [98]. Beyond *VEGF* regulation, arctigenin contributes to blood–retinal barrier preservation by upregulating tight junction proteins, including *occludin* and vascular endothelial cadherin (*VE-cadherin*). This effect is mediated through the suppression of matrix metalloproteinase-9 (MMP9) and TNFα, which otherwise cooperate to degrade endothelial junctional complexes [99,100,101,102,103]. Although Arctii Fructus lignans show strong in vitro aldose reductase inhibition, comparable to the therapeutic agent epalrestat at 200 μg/mL, their impact on the polyol pathway in DR has yet to be confirmed [104].

Current therapeutic approaches emphasize the cooperative role of VEGF and inflammatory mediators in the development of DR. While TNFα alone does not directly trigger retinal hyperpermeability, it amplifies VEGF-mediated vascular leakage through coordinated signaling [103,104,105]. The invasive nature and limitations of intravitreal anti-VEGF therapies highlight the necessity for multifunctional agents capable of targeting both mechanisms [106]. Arctii Fructus lignans, by modulating VEGF expression and inflammatory pathways while maintaining endothelial integrity, show potential as novel therapeutic candidates for DR. Further studies exploring their molecular interactions and tissue-specific pharmacokinetics may enable the design of noninvasive, multi-target treatment strategies.

## 4. Safety Profile and Pharmacological Basis for Clinical Application of Arctii Fructus Lignans in Diabetes

This section systematically synthesizes the in vivo processing rules and safety boundaries of the primary active components in Arctii Fructus (arctiin and arctigenin). It clarifies their absorption, distribution, metabolism, and excretion (ADME) characteristics, as well as toxicity risks, providing empirical support for clinical application.

In terms of pharmacokinetic properties, the in vivo behavior of Arctii Fructus lignans exhibits marked dependence on administration route and species-specific variability. For absorption and bioavailability, following subcutaneous injection of arctigenin into beagle *dogs* at doses of 0.134–1.209 µmol/kg, the compound was rapidly absorbed (time to peak concentration, T_max_ = 60–75 min) with high absolute bioavailability (F = 108% relative to intravenous injection). Peak plasma concentration (C_max_) showed a positive dose-dependent relationship: 0.032 ± 0.005 µmol/L at 0.134 µmol/kg and 0.252 ± 0.04 µmol/L at 1.209 µmol/kg. Sublingual administration (2.687–10.748 µmol/dog) yielded a bioavailability of 72.5% with T_max_ = 60–130 min, a route that avoids swallowing difficulties and is thus suitable for elderly or special patient populations [107]. In contrast, oral administration of arctigenin (2.687 µmol/kg) to Wistar rats resulted in extremely low plasma concentrations (most time points below the lower limit of quantification, LLOQ = 0.2 ng/mL). This is primarily attributed to intestinal first-pass metabolism: intestinal flora first hydrolyze arctiin to arctigenin, which is then catalyzed by UDP-glucuronosyltransferases (*UGTs*) to form inactive glucuronide conjugates [108]. A study of *piglets* receiving oral Arctii Fructus powder (1.0 g/kg·bw) further demonstrated that arctigenin was rapidly absorbed (T_max_ = 0.85 ± 0.21 h), widely distributed (apparent volume of distribution, V_d_ = 1.680 ± 0.402 L/kg), and slowly eliminated (elimination half-life, t_1/2_ = 63.47 ± 29.12 h). These findings confirm its ability to maintain therapeutic concentrations in vivo over extended periods, supporting its potential for long-acting treatment [109].

Regarding tissue distribution, following subcutaneous administration of arctigenin (0.806 µmol/kg) to rats, the drug rapidly distributed to all tested organs within 0.25–1 h. The highest concentration was detected in the intestine (0.67 ± 0.63 nmol/g at 0.25 h), followed by the liver (0.53 ± 0.15 nmol/g) and heart (0.52 ± 0.14 nmol/g). Moderate concentrations in the kidneys (0.23 ± 0.035 nmol/g) and pancreas (0.38 ± 0.078 nmol/g) provide anatomical support for its efficacy in improving diabetic nephropathy and protecting pancreatic β-cells. Notably, the drug crossed the blood–brain barrier, reaching a brain concentration of 0.077 ± 0.013 nmol/g at 0.25 h, suggesting potential benefits for diabetic neuropathy [107]. Species-specific differences in metabolism were further confirmed in liver microsome assays: after 90 min of incubation, the percentage of arctigenin remaining was highest in humans (62 ± 6.36%), followed by beagle *dogs* (25.9 ± 3.24%), *rats* (15.7 ± 9%), and *monkeys* (3.69 ± 0.12%). This indicates that human liver metabolizes arctigenin less efficiently, meaning higher plasma concentrations may be maintained at equivalent doses-necessitating targeted dose adjustments in clinical practice [107]. A study of mice receiving intravenous arctigenin (80 µg/mouse) also revealed that plasma concentrations of its major metabolite, arctigenin-4′-O-glucuronide (AGG), were significantly higher (4801.1 ± 105.7 ng/mL at 5 min) than those of the parent drug (1180.5 ± 283.0 ng/mL), confirming glucuronidation as the primary in vivo inactivation pathway [110]. Excretion pathway analysis showed that after subcutaneous injection of arctigenin (0.806 µmol/kg) to rats, cumulative urinary excretion over 72 h was only 1.94 ± 2.78%, fecal excretion over 48 h was 0.25 ± 0.21% (with enterolactone as the main metabolite, accounting for 35.80% of the administered dose), and biliary excretion over 12 h was 0.182 ± 0.141%. These data confirm that the drug is primarily eliminated via metabolism rather than direct excretion [107,111].

In toxicology, toxicity varied significantly across components, with clear dose and route dependence. The toxicity profile of pure arctigenin was well-characterized in a 28-day subcutaneous injection study in beagle dogs: at 60 mg/kg/day, animals developed severe damage to the lymphohematopoietic system (leukopenia, thrombocytopenia), digestive system (hepatocyte necrosis, cholestasis), urinary system (renal tubular degeneration), and cardiovascular system (myocardial hemorrhage), ultimately leading to the death of 5 dogs (3 females, 2 males). A dose of 20 mg/kg/day caused only mild lymphohematopoietic and digestive toxicity (no mortality), while 6 mg/kg/day produced no significant biochemical or pathological abnormalities. The no observed adverse effect level (NOAEL) for subcutaneous injection was determined to be <6 mg/kg, with no gender differences [112]. A 28-day chronic toxicity study of oral arctigenin in rats showed that 12 mg/kg/day induced focal necrosis of ventricular septal myocardium, mineralization of renal tubular epithelial cells, and elevated serum creatinine. At 36 mg/kg/day, more severe irreversible damage occurred, including bilateral testicular/epididymal atrophy, pulmonary edema, and vaginal epithelial dysplasia. Even 28 days after drug withdrawal, lesions such as myocardial necrosis and hepatocellular damage persisted, yielding a lowest observed adverse effect level (LOAEL) of 12 mg/kg for oral administration [113]. In contrast, Arctii Fructus fruit extract (ALFE) exhibited significantly lower toxicity: acute oral administration of ALFE (1000, 5000 mg/kg) to rats caused no mortality or abnormal behavior, with a median lethal dose (LD_50_) > 5000 mg/kg. Subchronic oral administration (300 mg/kg/day for 4 weeks) induced only mild pulmonary inflammation and small intestinal mucosal damage, with no abnormalities in hematological or biochemical markers. This reduced toxicity is speculated to stem from antagonistic effects of components like flavonoids in the extract, which may mitigate lignan-induced toxicity [114]. Additionally, long-term administration led to the accumulation of arctigenin in rat target organs (heart, liver, kidneys), a key contributor to persistent toxicity [115].

Integrated analysis of pharmacokinetic and toxicological data reveals that administration route directly impacts safety: while subcutaneous injection achieves high bioavailability, it carries substantial toxicity risks and is therefore not recommended for clinical use. In contrast, oral administration of arctiin or ALFE reduces bioavailability due to intestinal first-pass metabolism but concurrently lowers toxicity, making it more suitable for long-term adjuvant therapy. When combined with the recommended dose of arctiin granules (1.5 g/day) in Phase III clinical trials, the estimated arctigenin intake is far below the oral LOAEL (12 mg/kg/day) in rats, ensuring a sufficient safety margin. These findings not only clarify the in vivo processing and toxicity risks of Arctii Fructus lignans but also provide direct guidance for optimizing clinical administration routes (oral preferred), controlling doses (avoiding exceedance of 10 mg/kg/day), and monitoring the function of target organs (heart, liver, and kidneys).

## 5. Conclusions and Future Perspectives

This review consolidates the considerable preclinical evidence supporting the multi-targeted therapeutic potential of *Arctium lappa* L. fruit lignans, primarily arctiin and its active aglycone arctigenin, against diabetes mellitus and its complications. The pharmacological profile of these lignans is characterized by synergistic actions on glucose homeostasis, inflammation, oxidative stress, and cellular survival pathways—biological processes that are dysregulated in diabetes and its comorbidities—positioning these compounds as promising candidates for a holistic therapeutic approach that aligns with the complex pathophysiology of the disease. A critical insight emerging from the synthesized literature is the apparent limitation of their efficacy in advanced disease models with severe pancreatic β-cell dysfunction: preclinical data suggest that lignans excel at mitigating early-stage metabolic disturbances and preventing complication progression, rather than reversing end-stage tissue damage. This observation underscores a potentially crucial therapeutic window focused on early intervention, highlighting the need for future stratified clinical investigations that account for disease stage to maximize therapeutic benefit.

Despite the compelling bioactivity and preclinical promise, several translational challenges and fundamental questions must be addressed to advance these lignans toward clinical application. First, the pharmacological superiority of the naturally occurring lignan mixture, standardized per the Chinese Pharmacopeia (e.g., with a defined minimum arctiin content), over isolated compounds (such as pure arctigenin) remains a central, unproven hypothesis. Critically, toxicological comparisons reveal that the Arctii Fructus fruit extract (ALFE) exhibits a significantly improved safety profile compared to high-dose pure arctigenin, as detailed in Section 4. This empirical evidence provides a compelling pharmacological rationale for the traditional use and further investigation of the standardized extract over isolated compounds. The more favorable in vivo safety profile of the crude extract (compared to high-dose pure arctigenin) and the longstanding traditional use of the whole Arctii Fructus herb suggest potential synergistic interactions between lignans and other co-occurring phytochemicals (e.g., flavonoids), yet these interactions have not been systematically investigated. Second, the definitive structure–activity relationship (SAR) of these lignans—particularly the functional role of the glycosidic moiety (e.g., glucose) and the furan ring in mediating target binding (e.g., to α-glucosidase or adiponectin receptors) and biological activity—remains elusive. This knowledge gap hinders rational drug design efforts to optimize lignan potency or reduce off-target effects. Furthermore, the promise of a multi-targeted, naturally derived lignan mixture has not been rigorously tested against conventional single-target antidiabetic therapies (e.g., metformin, SGLT2 inhibitors) in well-designed clinical trials, leaving its comparative effectiveness and potential as an adjunctive treatment unvalidated.

Therefore, future research should be directed along several interconnected avenues to bridge these gaps. First, mechanistic and pharmaceutical optimization efforts are essential: employing integrated omics (e.g., transcriptomics, metabolomics) and chemical biology approaches will help delineate the precise molecular initiators of lignan action, clarify crosstalk between regulated pathways (e.g., *AMPK* and *NF-κB*), and unravel the basis for model-dependent variability in efficacy (e.g., differing responses in GK rats vs. db/db mice). Concurrently, developing advanced drug delivery systems, such as nanoformulations or lipid-based carriers, is imperative to overcome the inherent pharmacokinetic limitations of arctigenin, including its poor aqueous solubility and extensive intestinal first-pass metabolism, which currently restrict its in vivo bioavailability and therapeutic reach. Second, clinical translation and validation must be prioritized, with a focus on biomarker-guided, randomized controlled trials that utilize well-standardized lignan extracts. These trials should enroll patient cohorts stratified by diabetes type, disease stage, and complication profile (e.g., early insulin resistance vs. established nephropathy) to confirm the preclinical multi-target benefits in human populations, define the optimal therapeutic window (consistent with the early-intervention insight from preclinical studies), and objectively compare the efficacy and safety of the lignan mixture against standard-of-care drugs. Furthermore, investigating the pharmacological interplay within the lignan mixture and traditional Arctii Fructus formulations represents another key focus: understanding how individual lignans (and other phytochemicals) interact to enhance safety or potentiate efficacy will uncover the foundations of the mixture’s observed benefits, aligning with the holistic principle of traditional medicine that underpins the historical use of the whole herb.

In conclusion, while Arctii Fructus lignans offer a multifaceted and promising avenue for diabetes management, with preclinical data consistently demonstrating their ability to target the interconnected biological pathways driving diabetes and its complications, transforming this potential into evidence-based therapy necessitates a concerted, interdisciplinary effort. By bridging existing mechanistic gaps (e.g., defining SARs), addressing pharmaceutical challenges (e.g., improving delivery), and advancing rigorously designed clinical trials, researchers can translate the preclinical promise of these natural lignans into tangible, safe, and effective therapeutic options for patients living with diabetes and its associated complications.

## Figures and Tables

**Table 1:** The main lignan compounds in *Arctium lappa* L.

Number	Name	References
1	lappaol A	[11]
2	lappaol B	[12]
3	lappaol C	[12]
4	lappaol D	[13]
5	lappaol E	[14]
6	(+)-secoisolariciresinol	[15]
7	lappaol F	[16]
8	lappaol H	[16]
9	neoarctin A	[17]
10	neoarctin B	[18]
11	trachelogenin	[19]
12	diarctigenin	[20]
13	arctiinoside A	[21]
14	arctiinoside B	[21]
15	pinoresinol	[15]
16	matairesinol	[22]
17	arctignan A	[21]
18	arctignan G	[21]
19	arctignan H	[21]
20	(+)-7,8-didebydroarctigenin	[23]
21	Arctiin	[23]
22	Arctigenin	[23]

## Data Availability

No new data were created or analyzed in this study. Data sharing is not applicable to this article.
